# Subclavian chronic total occlusion angioplasty facilitates cardiac implantable electronic device upgrades: A single-center case series

**DOI:** 10.1007/s10840-026-02320-y

**Published:** 2026-04-13

**Authors:** Richard Amoateng, Emmanuel Olumuyide, Xiarepati Tieliwaerdi, Sruti Prathivadhi-bhayankaram, Sukit Ringwala, Dana Johnson, Joaquim Spadoni Barboza

**Affiliations:** 1 Division of Cardiology, University of Illinois, Chicago, IL USA; 2 Division of Internal Medicine, Advocate Masonic Medical Center, Chicago, IL USA; 3 Department of Internal Medicine, Allegheny General Hospital, Pittsburgh, PA USA; 4 Department of Cardiology, Northwestern University Feinberg School of Medicine, Chicago, IL USA

**Keywords:** Subclavian venoplasty, Chronic total occlusion, Cardiac resynchronization therapy, Venous access, Lead revision

## Abstract

**Background:**

Subclavian venous occlusion is a frequent complication of transvenous cardiac implantable electronic device (CIED) implantation, with reported incidences exceeding 30%. Although often asymptomatic, chronic total occlusion (CTO) creates a major technical barrier during device revision, particularly for cardiac resynchronization therapy (CRT) upgrades. Conventional strategies such as lead extraction or contralateral tunneling may increase procedural risk or compromise future vascular access. Subclavian CTO venoplasty is a minimally invasive alternative to re-establish ipsilateral access.

**Methods:**

We describe a single-center series of six patients (ages 62–78 years) who underwent percutaneous subclavian, axillary, or brachiocephalic venoplasty to facilitate CIED upgrade or lead revision. Our approach favored ipsilateral upper-extremity access (typically basilic vein) with a 0.018-inch platform using polymer-jacketed guidewires and microcatheters for CTO crossing. Serial balloon angioplasty (4.0–10.0 mm; 40–80 mm length range) was performed using stepwise inflation (nominal pressure with escalation as needed up to rated burst pressure per device specifications) to restore venous patency.

**Results:**

Technical success was achieved in all six cases (100%), enabling four CRT implantations, one left bundle branch area pacing (LBBAP) implant, and one dual-chamber lead revision. No major intraprocedural complications occurred; one patient developed a self-resolving chest wall hematoma. Procedure time ranged from 116 to 240 min. Among CRT recipients, QRS duration narrowed (mean reduction 45 ± 18 ms). Follow-up (2–12 months) demonstrated stable lead parameters and no clinical evidence of recurrent occlusion.

**Conclusion:**

In this small series, subclavian/central venous CTO venoplasty using a brachial-first, 0.018-inch CTO-oriented strategy was feasible and enabled successful ipsilateral CIED revision while preserving vascular access. Larger studies are needed to better define the success rate and safety relative to the extraction or subclavian/device pocket access only approach.

**Supplementary Information:**

The online version contains supplementary material available at 10.1007/s10840-026-02320-y.

## Introduction

The prevalence of cardiac implantable electronic devices (CIEDs) continues to rise in an aging population, leading to an increasing need for system upgrades and revisions. A well-recognized sequela of transvenous pacing is subclavian or axillary venous stenosis/occlusion, with incidence rates reported as high as 30% [1, 2]. While often clinically silent due to collateral formation, venous occlusion can present a challenge when ipsilateral access is required for cardiac resynchronization therapy (CRT) upgrades or lead revisions [3].

Traditional strategies to overcome occluded access include transvenous lead extraction, contralateral tunneling, or surgical alternatives. However, extraction carries risks such as vascular tear and tamponade, particularly with long lead dwell time [4, 5]. Tunneling preserves the existing system but may increase lead burden, mechanical stress, and limit future venous access [6, 7].

Percutaneous venoplasty offers a less invasive alternative, restoring patency to chronically occluded segments using techniques adapted from complex peripheral and coronary CTO intervention, including microcatheter-supported wiring and controlled balloon dilation [8]. Prior large series (e.g., Worley et al.) established feasibility at scale [9]. The focus of the present report is to provide a practical, contemporary technical framework emphasizing a brachial-first, 0.018-inch CTO-oriented crossing algorithm, explicit wire escalation/de-escalation strategy, and troubleshooting maneuvers applicable to electrophysiology-led device revision workflows.

## Methods and venoplasty technique

### Patient selection

This retrospective series includes six consecutive patients presenting with symptomatic bradycardia or heart failure requiring CIED upgrade or revision who were found to have complete subclavian, axillary, brachiocephalic, and/or superior vena cava (SVC) occlusion.

### Access strategy

We preferentially obtain ultrasound (US)-guided ipsilateral upper-extremity venous access using a 6 French (Fr) slender sheath. The basilic vein is preferred due to its favorable trajectory into the axillary/subclavian system; the cephalic vein is avoided as its deltopectoral angulation limits support. Femoral venous access is not routine and is reserved as a bailout for (i) bidirectional crossing, (ii) snaring/externalization, (iii) improved delineation of proximal/distal caps, or (iv) complication management.

### Venogram assessment and decision process

Digital subtraction angiography (DSA) with breath-hold is performed to define: (i) the proximal and distal caps, (ii) the apparent occlusion length, (iii) inflow/outflow quality, and (iv) collateral anatomy. Collateral presence typically suggests chronicity and helps localize the target course, but does not, by itself, exclude venoplasty when ipsilateral venous access is clinically required for device revision. We recognize that angiographic length can overestimate the true fibrotic segment, especially in the presence of dense collaterals, and a bidirectional strategy may be used when anatomy is ambiguous.

### Occlusion length considerations

There is no absolute occlusion length cutoff. However, longer or more ambiguous occlusions (e.g., spanning the brachiocephalic vein or involving the SVC) more often prompt early consideration of dual access (upper extremity plus femoral) to confirm intraluminal position and facilitate snaring/externalization when needed.

### CTO crossing strategy (0.018-inch platform)

We use a microcatheter-supported, stepwise escalation approach:

Primary platform (preferred):

- Microcatheter: 0.018-inch compatible microcatheter (angled tip preferred); Navicross Angled (Terumo) or Trailblazer Angled support catheter (Medtronic) for directional control and support.
- Primary wire (polymer-jacketed): Gladius Mongo 0.018-inch (Asahi) or V18 Control (Boston Scientific). These are selected for trackability and controlled knuckling, which can reduce perforation risk when navigating uncertain tissue planes.

### Escalation (penetration)

If the polymer-jacketed wire cannot cross a resistant proximal cap, escalation is performed to a stiff, tapered, non-polymer penetrative CTO wire (e.g., Astato 20/40 [Asahi], Hornet 14 [Boston Scientific], or Gaia series [Asahi]) with frequent orthogonal fluoroscopic checks. Existing leads are used as an anatomic reference to maintain the expected venous course.

### De-escalation (safety)

Once the occlusion cap is penetrated and a channel is established, we exchange back to a polymer-jacketed wire to reduce the risk of perforations, as polymer-jacketed wires tend to follow vessel structure.

### Confirmation of intraluminal position

Intraluminal positioning is confirmed by wire/microcatheter passage into the right atrium or inferior vena cava (IVC) in at least two orthogonal views.

### Balloon Angioplasty (diameter, length, and inflation approach)

Balloon angioplasty is performed serially using low-profile 0.018-inch balloons. Typical diameters range 4.0–10.0 mm with balloon lengths commonly 40–80 mm, selected to cover the fibrotic segment while minimizing dilation of the adjacent normal vein.

Inflation is performed in a controlled stepwise manner:

- initial inflation at nominal pressure,
- incremental increases if a persistent waist remains,
- without exceeding the rated burst pressure (RBP) specified for the balloon.

### Lead implantation and re-entry to the pocket

Following successful venoplasty, the device pocket is opened. When direct wire externalization is not feasible, balloon-assisted puncture can be performed by targeting the inflated balloon within the recanalized segment, followed by standard lead implantation techniques.

### Contraindications and safety considerations

Absolute contraindication: active CIED infection requiring extraction.

Relative contraindications: uncontrolled coagulopathy, inability to tolerate procedural anticoagulation/contrast, severe frailty where procedural benefit is limited, or anatomy where safe intraluminal confirmation cannot be achieved.

Perforation/dissection bailout plan: Wire exits in general are done by stiff wires (0.014 in our algorithm) and are non-consequential if not followed by larger equipment (e.g., balloon). If venous perforation is suspected (contrast extravasation or hemodynamic concern) and the CTO has been crossed, we maintain wire access and perform immediate balloon tamponade at the suspected site with prolonged low-pressure inflation. Anticoagulation is reversed when appropriate. Escalation to a covered stent and/or surgical consultation is rare.

Lead burden considerations: The number of existing leads and the presence of abandoned leads are assessed. High lead burden does not automatically preclude venoplasty, but it may shift the strategy toward extraction or contralateral options if anticipated crowding prevents sheath passage or long-term access preservation.

### Approach to central venous/SVC occlusion

When occlusion involves the brachiocephalic vein or SVC, often near defibrillator coil segments, dual access (upper extremity and femoral) might be necessary to improve support, and angle of attack for the CTO segment. Externalization of the wire can further increase support for balloon uncrossable lesions. If recanalization cannot be achieved safely, alternative strategies include extraction at experienced centers, contralateral implantation with tunneling, epicardial lead placement, conduction system pacing alternatives, or leadless pacing, depending on clinical goals.

## Case Presentations

### Case 1: Biventricular upgrade via brachial approach

A 69-year-old male with a mechanical aortic valve and pacing-induced cardiomyopathy (left ventricular ejection fraction 35%) presented for upgrade to biventricular pacing. Venography demonstrated total left subclavian occlusion. Via a left brachial approach, the CTO was crossed using an angled microcatheter and a 0.018-inch Gladius Mongo wire (Video 4). Following 5.0 mm balloon angioplasty, a quadripolar left ventricular lead was successfully placed. The QRS duration narrowed from 214 to 164 ms with clinical improvement.

Device selection note: CRT-P versus CRT-D was determined by individualized sudden cardiac death risk assessment and shared decision-making in the clinical context, including patient preference.

### Case 2: Rescue of access for CRT-D

A 62-year-old male with nonischemic cardiomyopathy and high right ventricular (RV) pacing thresholds required a CRT-defibrillator (CRT-D) upgrade. Access was complicated by the complete occlusion of the left subclavian vein and superior vena cava (SVC). A bidirectional approach was used with femoral venous access to support snaring/externalization attempts and improve visualization of caps. The lesion was successfully crossed using a 4F glide angled tip catheter (Terumo) and a V18 Control (Boston Scientific) guidewire. It was then dilated serially up to 9.0 mm with the PTA Evercross 9 × 80 mm × 80 cm Balloon (Medtronic) (video 2). New RV and left ventricular leads were implanted successfully and connected to a CRT-D pulse generator (model details omitted for clarity). The patient developed a self-limiting chest wall hematoma without the need for intervention.

### Case 3: LBBAP upgrade with AV node ablation

A 75-year-old male with permanent atrial fibrillation and RV dysfunction required an upgrade and atrioventricular (AV) node ablation. After unsuccessful standard access, a left brachial approach was used to cross a subclavian occlusion with a Gladius Mongo wire. Wire escalation was performed using Hornet 14 guidewire (Boston Scientific) but eventually crossed with Gladius Mongo 0.018-inch (Asahi Intec). Given RV dysfunction, conduction system pacing was selected. A lumen-less lead (Medtronic 3830) was deployed in the left bundle branch area, achieving a narrow-paced QRS.

### Case 4: Complex anatomy and thebesian valve challenge

A 72-year-old male with ischemic cardiomyopathy underwent CRT-D upgrade while on guideline-directed medical therapy (GDMT). The subclavian CTO was crossed -supported technique and dilated using the 0.018-inch Trailblazer Angled support catheter (Medtronic) and Gaia Next 3 PTCA (Asahi Intec). A prominent Thebesian valve complicated coronary sinus (CS) access. Using a telescoping sheath strategy and a polymer-jacketed wire (Sion Blue), the valve was negotiated, and the left ventricular lead was deployed in a basolateral branch.

### Case 5: Axillary vein CTO

A 78-year-old male presented for lead revision due to a right atrial (RA) lead fracture. Venography revealed an axillary vein CTO. An angled microcatheter (4F glide angled tip catheter [Terumo]) and a stiff penetrative wire (Astato Xs20 [Asahi] were required to engage the proximal cap, followed by exchange to a polymer-jacketed wire (Gladius Mongo 0.018-inch [Asahi]) and 5.0 mm balloon angioplasty (Video 7). Patency was restored, enabling the successful implantation of a new RA lead.

### Case 6: CRT-P upgrade with his bundle pacing

A 74-year-old male with pacing-induced cardiomyopathy required an upgrade. Venography confirmed a brachiocephalic vein CTO. The lesion was resistant to polymer-jacketed wires and was crossed with a penetrative CTO wire (Gaia 2[Asaha Intec]) via a 2.1F Mamba Flex microcatheter (Boston Scientific). Following serial dilation, a His bundle sheath was advanced, and a standard stylet-driven lead was fixed to the septum. Left ventricular function improved from 35 to 55% at 9 months.

## Discussion

This series illustrates that subclavian/central venous CTO venoplasty can enable complex CIED revision while preserving ipsilateral access. Using CTO-derived techniques, particularly a brachial-first vector and a 0.018-inch microcatheter-supported wiring strategy, we achieved technical success in six consecutive cases without major intraprocedural complications.

### Comparison with alternatives

Venous occlusion forces a procedural decision matrix. Lead extraction is definitive but carries the highest risk profile, including major vascular injury, particularly with long dwell time or dense fibrosis [4, 5]. Contralateral tunneling avoids crossing the occlusion but increases lead burden and may limit future venous options [6, 7]. In contrast, venoplasty preserves the original access side and can be performed as an endovascular strategy. Worley et al. established feasibility at scale [9]; our series complements this work by providing a structured CTO-oriented workflow emphasizing 0.018-inch platforms, escalation/de-escalation strategy, and practical re-entry maneuvers relevant to contemporary upgrade scenarios.

### Technical nuances

The brachial/upper-extremity approach provides a straight trajectory for force transmission, often improving control compared with working from the pocket angle alone. We found that many fibrotic occlusions respond better to a stepwise 0.018-inch strategy than to traditional 0.035-inch hydrophilic wiring. We also address central venous and SVC disease by incorporating dual access and snaring/externalization when needed.

### Lead burden and central occlusion

Lead number and abandoned leads are important considerations. In our experience, “crowding” is not an absolute barrier if the occluded segment can be safely dilated and sheath passage is feasible; however, extreme lead burden or extensive SVC involvement may favor extraction at experienced centers or alternative systems, depending on patient goals and anatomy.

### Limitations

**Fig. 1:**
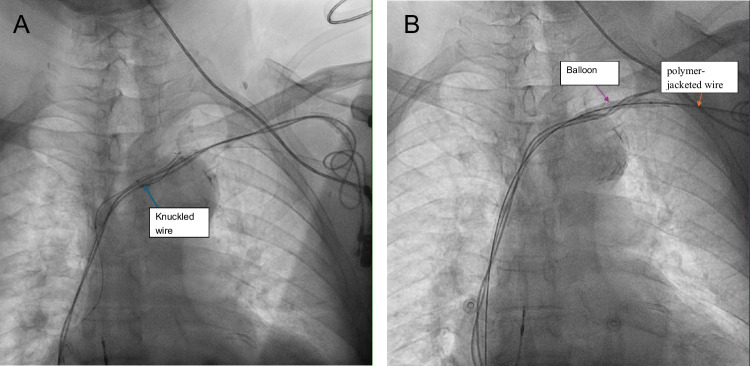
**A** Angiogram showing knuckled wire **B** Angiogram showing balloon with polymer-jacketed wire.

This is a single-center retrospective series with a small sample size and no control group. Therefore, while our cases demonstrate feasibility and a low observed complication rate, definitive safety conclusions cannot be drawn from six patients alone. Our findings should be interpreted as a technical complement to larger published experiences, and larger prospective studies are needed to compare outcomes, complications, and long-term patency relative to extraction or contralateral approaches. Fig. 1

## Conclusion

Subclavian/central venous CTO venoplasty using a brachial-first, 0.018-inch CTO-oriented strategy can restore ipsilateral venous access and facilitate complex CIED upgrades and revisions while preserving future access options. In this small series, the approach was feasible with low observed complications; larger studies are needed to better define comparative safety and durability.

## Supplementary Information

Below is the link to the electronic supplementary material.

Supplementary file1 (MOV 8325 KB)

Supplementary file2 (MOV 8032 KB)

Supplementary file3 (MOV 13563 KB)

Supplementary file4 (MOV 18485 KB)

Supplementary file5 (MOV 883 KB)

Supplementary file6 (MOV 814 KB)

Supplementary file7 (MOV 1877 KB)

## Data Availability

No datasets were generated or analysed during the current study.
